# Crosstalk between hypoxia-inducible factor-1α and short-chain fatty acids in inflammatory bowel disease: key clues toward unraveling the mystery

**DOI:** 10.3389/fimmu.2024.1385907

**Published:** 2024-03-28

**Authors:** Jinyin Xiao, Xiajun Guo, Zhenquan Wang

**Affiliations:** ^1^ Graduate School, Hunan University of Traditional Chinese Medicine, Changsha, China; ^2^ Department of Anorectal, the Second Affiliated Hospital of Hunan University of Traditional Chinese Medicine, Changsha, China; ^3^ Department of Geriatric, the First People’s Hospital of Xiangtan City, Xiangtan, China

**Keywords:** inflammatory bowel disease, hypoxia-inducible factor-1α, short-chain fatty acids, intestinal epithelial barrier, gut immunity

## Abstract

The human intestinal tract constitutes a complex ecosystem, made up of countless gut microbiota, metabolites, and immune cells, with hypoxia being a fundamental environmental characteristic of this ecology. Under normal physiological conditions, a delicate balance exists among these complex “residents”, with disruptions potentially leading to inflammatory bowel disease (IBD). The core pathology of IBD features a disrupted intestinal epithelial barrier, alongside evident immune and microecological disturbances. Central to these interconnected networks is hypoxia-inducible factor-1α (HIF-1α), which is a key regulator in gut cells for adapting to hypoxic conditions and maintaining gut homeostasis. Short-chain fatty acids (SCFAs), as pivotal gut metabolites, serve as vital mediators between the host and microbiota, and significantly influence intestinal ecosystem. Recent years have seen a surge in research on the roles and therapeutic potential of HIF-1α and SCFAs in IBD independently, yet reviews on HIF-1α-mediated SCFAs regulation of IBD under hypoxic conditions are scarce. This article summarizes evidence of the interplay and regulatory relationship between SCFAs and HIF-1α in IBD, pivotal for elucidating the disease’s pathogenesis and offering promising therapeutic strategies.

## 1 Introduction

Inflammatory Bowel Disease (IBD), encompassing Crohn’s Disease (CD) and Ulcerative Colitis (UC), represents chronic intestinal disorders characterized by recurrent inflammation. The annual surge in IBD prevalence marks it a major public health issue worldwide, across developed and developing countries alike (1). Factors contributing to IBD include genetic predisposition, diet, lifestyle, environmental factors, psychological stress, gut microbiota, and host immunity (2, 3). IBD pathology is primarily characterized by impaired intestinal epithelial barrier (IEB) function, significant immune dysregulation, and gut microbiome disturbances (4, 5). These pathologies are interrelated rather than isolated, with IEB impairment leading to heightened exposure of mucosal immune cells to luminal antigens and inflammatory mediators, as well as to gut microbiota and their metabolites. This exposure strains intestinal defenses, including the IEB and mucosal immunity, disrupting homeostasis and intensifying inflammation (6–9). Therefore, IEB integrity, immune function, and gut microbiome are critical in the pathogenesis and management of IBD and remain focal points in current and future research endeavors.

Currently, IBD is mostly incurable, focusing on symptom management and relapse prevention, sometimes requiring surgery. Conventional treatments are pharmacological, including various drugs like antibiotics, aminosalicylates, corticosteroids, immunomodulators, and biologics (10), but these have many limitations (11). Although many novel therapies, such as cell therapies and small-molecule drugs, are constantly being discovered, they are still in clinical trials with unclear risks (10, 12). Therefore, elucidating the mechanisms and links between the aforementioned pathological factors in IBD could enhance treatment strategies.

Hypoxia-inducible factor-1 (HIF-1), a key transcription factor during the hypoxic response, comprises two subunits, HIF-1α and HIF-1β (13). HIF-1α plays a broad physiological role, activating target genes to regulate body’s hypoxic and inflammatory responses, crucial for cell adaptation to hypoxia and inflammation (14, 15). Additionally, HIF-1α is central to regulating the integrity and permeability of IEB (16, 17). Hence, HIF-1α is vital for IEB function, gut immunity, and pathogen defense, marking it as a key target in treating inflammatory diseases like IBD. Short-chain fatty acids (SCFAs) are mainly fermented from dietary fiber by intestinal microorganisms like Firmicutes and Bacteroidetes, which mainly include acetate, propionate, and butyrate, which account for more than 95% (18–20). Recent studies have validated the significant physiological and pathological roles of SCFAs in maintaining IEB function, modulating gut immunity, and sustaining microbial homeostasis (21). Consequently, dysregulation of gut metabolites, especially SCFAs, is considered a key factor in causing IBD-related intestinal inflammation and IEB dysfunction (22). During IBD development, HIF-1α is also regulated by SCFAs, with complex pathways and effects, particularly on IEB function and gut immunity. Therefore, this article details the role and mechanism of SCFAs in regulating HIF-1α in IBD, aiming to provide new strategies and avenues for clinical treatment.

## 2 Gut and hypoxia

### 2.1 Basic overview of hypoxia

Normoxia and hypoxia represent two distinct oxygen concentration states in cellular environments (23). Normoxia denotes the normal oxygen levels in organs, tissues, and cells in healthy states, with an oxygen partial pressure of 10%-20% (23, 24). In this state, oxygen supply is sufficient for bodily functions without restriction. However, hypoxia arises when oxygen supply falls short of demand, resulting in subnormal levels, often under 5% oxygen partial pressure (23, 25). Nonetheless, hypoxia does not always indicate pathology. It occurs under normal physiological conditions as well, thus hypoxia is classified into physiological and pathological types (26, 27).

Physiological hypoxia is influenced by factors such as diet (28), exercise (29), and living environment (such as at high altitude) (30). The body triggers regulatory and adaptive responses to mitigate oxygen deficiency, restore hypoxic conditions for oxygen homeostasis, or boost cellular tolerance to hypoxia. For instance, the body may enhance cardiac output or elevate erythropoiesis to augment tissue oxygenation, processes intimately associated with HIF (30–32). Additionally, pathways such as HIF signaling are employed for tissue cell acclimatization to hypoxia (33). It’s critical to recognize that hypoxia is not inherently detrimental. In certain areas, such as the gastrointestinal tract, moderate and stable physiological hypoxia is essential for maintaining key physiological processes like immune homeostasis, cellular metabolism, and angiogenesis (34, 35). In contrast, pathological hypoxia, a common feature in many diseases, is more prevalent and severe, playing a vital role in the pathology of cardiac (36), pulmonary (37), autoimmune diseases (38), and cancer (39). Importantly, pathological hypoxia is significantly linked to physiological hypoxia. Disruption of physiological hypoxia can initiate a series of cellular alterations, precipitating pathological hypoxia (27). Both hypoxia types are modulated by HIF, with IBD research centering on gut physiological hypoxia. Therefore, understanding the role and mechanism of HIF-related signaling pathways is crucial for treating hypoxia-associated diseases, including IBD.

### 2.2 Intestinal hypoxia condition

Relative hypoxia is a normal physiological condition for certain organs and tissues. The gastrointestinal tract, influenced by its anatomical structure, complex vascular system, and the distribution and metabolism of the microbiota, naturally forms steep oxygen gradients both horizontally and vertically, with extensive research conducted in the colon (40–42). Studies have confirmed a gradient decrease in oxygen tension from the stomach to the intestines, with gastric luminal oxygen tension at about 7.7 kPa, small intestinal luminal tension at 4.2 - 4.6 kPa, and colonic tension dropping to 0.4 - 1.5 kPa (43–45). Interestingly, a near-vertical oxygen tension drop is noted from various intestinal wall layers to the lumen, where muscularis mucosae holds much higher tensions than the lumen. Colonic muscle oxygen levels range from 5.6 to 9.5 kPa, the submucosa around 5.6 kPa, and the luminal mucosa surface between 0.13 to 0.8 kPa (34, 46–50). Thus, hypoxia and oxygen gradients are crucial physiological features of the gut, with disruption potentially leading to disease.

Notably, the established oxygen gradients are closely associated with HIF-1α and the spatial distribution of intestinal microbiota. Studies have indicated that in the oxygen-rich stomach and duodenum, aerobic and facultative anaerobic bacteria are dominant; in the small intestine, facultative anaerobes are most common; and in the oxygen-depleted large intestine, obligate anaerobes prevail (34, 51–53). These findings suggest that microbiota distribution critically influences intestinal oxygen gradients, as evidenced by germ-free mouse studies. Kelly et al. reported that antibiotics or germ-free conditions significantly reduce SCFAs levels, diminish HIF-1α in intestinal epithelial cells (IEC), and nearly eliminate physiological hypoxia and oxygen gradients (54). Wang et al. also discovered that gut microbiota and SCFAs, especially butyrate, are intimately linked to intestinal hypoxia; butyrate enhances oxygen consumption and stabilizes HIF via prolyl hydroxylase enzymes (PHD) inhibition (55). Additionally, the formation of intestinal oxygen gradients is also related to other metabolic products such as trimethylamine oxide and indole derivatives, with antibiotic exposure altering these processes (49). Research by Nicolas et al. validated these points (56). In summary, HIF-1α and/or the gut microbiota and their metabolites, SCFAs, are key factors in the formation of intestinal hypoxia.

### 2.3 HIF-1α in hypoxia

**Figure 1:**
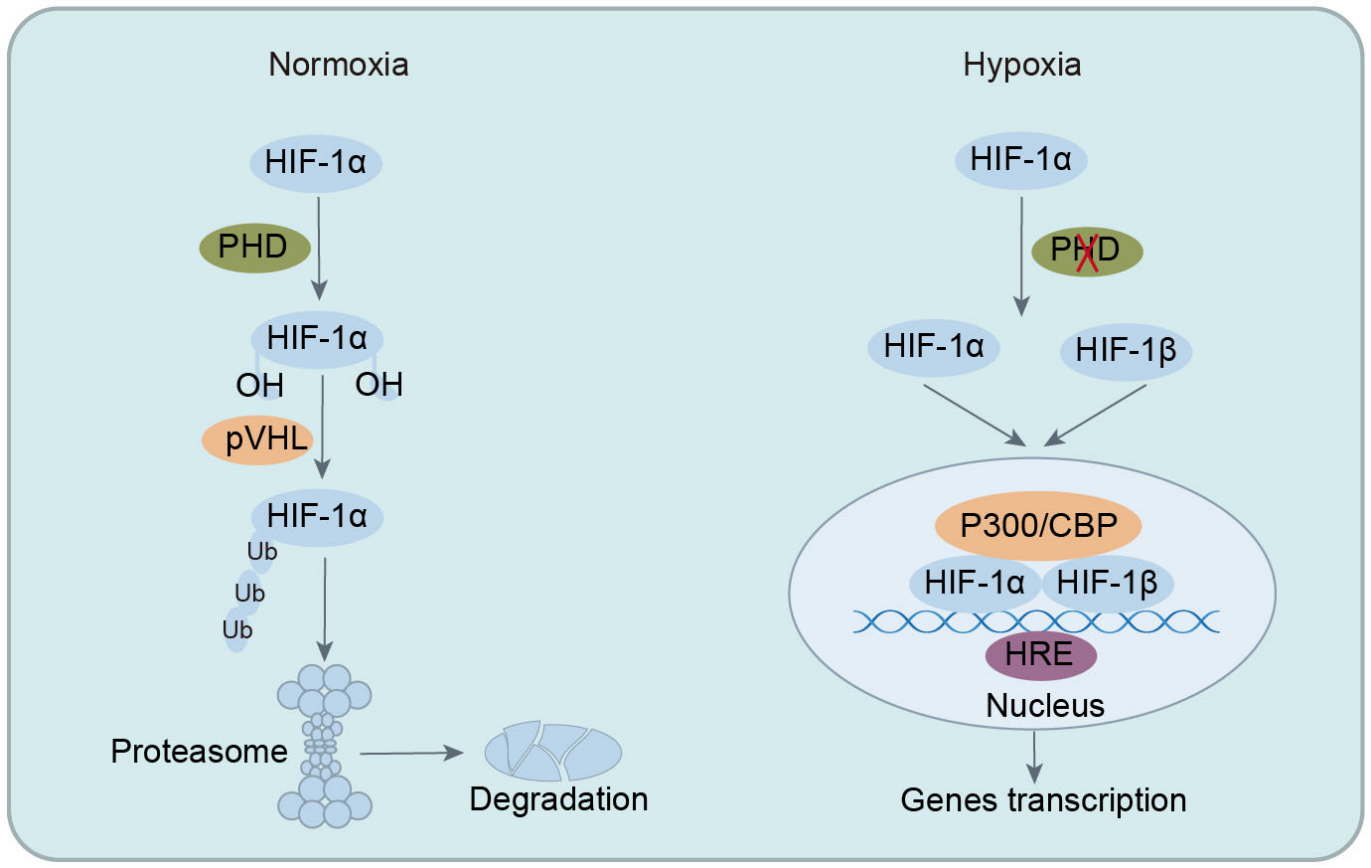
Regulation of HIF-1 stability under normoxia and hypoxic conditions. In normoxia, HIF-1α is hydroxylated by PHD, leading to ubiquitination and degradation, preventing its biological function. In hypoxia, ubiquitination of HIF-1α is inhibited, allowing its translocation into the nucleus to form heterodimeric complexes with HIF-1β. This complex then binds to specific DNA regions of target genes, activating the transcription of numerous downstream targets. HIF-1α, hypoxia-inducible factor-1α; PHD, prolyl hydroxylase enzymes; VHL, von Hippel-Lindau; CBP, CREB-binding protein; HRE, hypoxia-response elements.

HIF functions as a heterodimer composed of α and β subunits, primarily including HIF-1 (HIF-1α and HIF-1β), HIF-2 (HIF-2α and HIF-2β), and HIF-3 (HIF-3α and HIF-3β) (57, 58). Among these types, HIF-1 is the most thoroughly researched and is vital for hypoxic pathophysiology. HIF-1 is ubiquitous in tissues and cells, with HIF-1α expression regulated by oxygen levels and HIF-1β expressed continuously (44). HIF-1 exerts its extensive and potent biological functions only when HIF-1α and HIF-1β combine to form a heterodimeric structure. Under normoxia, HIF-1α, hydroxylated by oxygen-dependent PHD, binds to von Hippel-Lindau (VHL) protein, which recruits ubiquitin ligases to HIF-1α, leading to its proteasomal degradation and functional inactivity. However, in hypoxia, the absence of oxygen impedes the ubiquitination of HIF-1α, enabling its nuclear translocation to form a heterodimeric complex with HIF-1β. This complex then binds to specific DNA regions (hypoxia-response elements, HRE) of target genes, activating the transcription of numerous downstream genes. This activation plays a crucial role in processes such as cellular metabolism, intestinal barrier function, immune regulation, angiogenesis, erythropoiesis, tumorigenesis, cell apoptosis, and autophagy (33, 59, 60). In summary, HIF-1α plays a pivotal role in maintaining cellular oxygen homeostasis under hypoxic conditions and offers significant protection during pathological and physiological hypoxia by mediating adaptive responses to oxygen insufficiency (**Figure 1**).

## 3 HIF-1α in IBD

### 3.1 Hypoxia/HIF-1α and inflammation in IBD

Inflammatory states are considered a key pathological feature of IBD, while hypoxia is seen as a common characteristic of most inflammation. Research has confirmed significant hypoxia in IBD’s inflamed regions, which mutually affects each other, with HIF-1α being central to the connection between host hypoxia and inflammation (44). Current consensus in research posits physiological hypoxia as a crucial environmental characteristic of the gut, with IBD pathogenesis closely linked to disruptions in this hypoxic milieu. A stable hypoxic environment effectively controls and alleviates intestinal inflammation, closely linked to the activation of HIF-1α, which promotes adaptation to hypoxia, mitigates gut inflammation, and maintains intestinal barrier, serving a definitive protective role in IBD (48). For example, moderate hypoxia preserves the intestinal barrier in mouse dextran sulfate sodium (DSS)-induced colitis via HIF-1α modulation of Vitamin D Receptor (VDR) signaling (61). Additionally, hypoxia significantly reduces inflammatory gene expression in CD patients and colitis mice, linked to the inhibition of the NOD-like receptor thermal protein domain associated protein 3 (NLRP3)/mammalian target of rapamycin (mTOR) pathway and autophagy induction by hypoxia-driven HIF-1α (62). Furthermore, in colitis mouse models, butyrate upregulation activates IEC peroxisome proliferator-activated receptor γ (PPAR-γ), reducing cellular oxygenation in inflammation, boosting mitochondrial function and β-oxidation of SCFAs to sustain hypoxia and curb pathogenic bacterial growth in IBD (63–65). Conversely, deficiency or impairment of HIF-1α in the gut exacerbates intestinal inflammation. Myeloid HIF deficiency in DSS-induced colitis leads to increased infiltration of neutrophils and Ly6C monocytes, with a more marked impact from HIF-1α loss compared to HIF-2α (66).

Interestingly, research has indicated hypoxia induces or exacerbates inflammation, with HIF-1α activation contributing to IBD’s inflammatory processes. For instance, frequent high-altitude activities have been associated with gastrointestinal inflammation, increasing the risk of IBD flares (67). Hypoxia markers HIF-1α, macrophage inflammatory protein 3α (MIP-3α), and vascular endothelial growth factor (VEGF) were significantly elevated in colonic epithelial tissues and serum of both pediatric and adult patients with active IBD, correlating positively with histological severity (68). In DSS-induced colitis mice, myeloid HIF-1α deletion lessened inflammation, HIF-2α deletion worsened it, and the absence of both showed no significant changes (69); however, increased myeloid expression of HIF-1α accelerated colitis progression (70). Moreover, hypoxia was found to not only exacerbates DSS-induced colitis but also augments neuroinflammation in mice (71). However, the specific mechanisms and pathways by which hypoxia/HIF-1α induces or exacerbates intestinal inflammation have not been elucidated in these studies, necessitating further research for clarification. It is worth noting that inflammation also aggravates hypoxia. In the pathology of IBD, prolonged intestinal inflammation sharply increases the severity of mucosal hypoxia, potentially transitioning to a chronic pathological state (44). This exacerbates intestinal damage and perpetuates a vicious cycle. In IBD, the exacerbation of local hypoxia by intestinal inflammation may be associated with the following factors. On the one hand, inflammatory recurrence in IBD leads to vascular damage, endothelial dysfunction, and reduced perfusion (72, 73), while chronic inflammation increases oxygen consumption due to immune cell infiltration, exacerbating hypoxia (7, 74, 75). On the other hand, changes in the gut microbiota and their metabolites also consume oxygen, with obligate anaerobes switching to facultative anaerobes and less butyrate production, disrupting physiological hypoxia (76). Additionally, anaerobic bacteria metabolites enhance the consumption of oxygen in IEC, maintaining gut hypoxia (77–79). Hence, intestinal hypoxia is considered a common microenvironmental feature of IBD pathology, with inflammatory damage spreading hypoxia to deeper layers like the submucosa, exacerbating gut hypoxia.

Certainly, in the face of hypoxia, inflammation is not passive. In UC, it prompts a transcriptional reprogramming of the HIF pathway, downregulating PHD1 to favor HIF-1α’s protective role over HIF-2’s harmful effects, aiding mucosal repair (80). This highlights the close relationship between HIF-1α and the protective mechanisms against inflammatory hypoxia in IBD. Notably, different subtypes of HIF play distinct roles in hypoxia and the pathogenesis of IBD. HIF-1 is primarily involved in acute hypoxic response, whereas HIF-2 and HIF-3 are implicated in chronic hypoxia adaptation (33, 58). There is also cross-talk among these subtypes. A decrease in HIF-1 levels leads to an increase in HIF-2 and HIF-3, a shift reminiscent of cell polarization, which is necessary for the long-term hypoxic adaptation of cells (58). Moreover, in IBD, different HIF subtypes exert critical but opposing effects. As mentioned earlier, HIF-1α suppresses intestinal inflammation and promote resolution in IBD. Conversely, HIF-2α activation upregulates inflammatory expression, intensifying the inflammatory response, aggravating IBD, and is associated with colorectal cancer (CRC) progression (81–84).

Although the impact of hypoxia in IBD is contested, a stable hypoxic environment is pivotal for gastrointestinal wellness, with HIF-1α’s protective role acknowledged. HIF-1α orchestrates cellular adaptation to hypoxic and inflammatory conditions via downstream genes regulation, essential for cells coping with such stresses. Therefore, therapeutic approaches for IBD have diversified to include tactics like PHD inhibition to manage HIF-1α, enhancing treatment options (85, 86). The debate surrounding hypoxia’s pro-inflammatory or anti-inflammatory effects in IBD may hinge on varying experimental hypoxia parameters (the level, duration, and degree of hypoxia), HIF-1α’s cell-specific roles, the target genes or pathways affected by HIF-1α, and interactions between HIF isoforms. Thus, in exploring HIF’s role or therapeutic use in IBD, these considerations are essential.

### 3.2 The role and mechanism of HIF-1α in IBD

#### 3.2.1 HIF-1α regulation of intestinal barrier

The IEB, comprising IEC and the basement membrane, serves as a vital physiological barrier in the gut, essential for nutrient digestion and absorption, preserving gut hypoxia, and preventing luminal waste, microbiota, and allergens from entering (87, 88). Dysfunction of the IEB leads to intestinal damage, inflammation, and even sepsis and bacteremia (89). Therefore, the IEB plays a pivotal role in the pathology of IBD. IEC are primary targets of hypoxia, situated between the hypoxic lumen and the well-perfused lamina propria. Under physiological conditions, IEC are hypoxia-tolerant, aiding gut adaptation to low oxygen and maintaining IEB integrity (88, 90). The hypoxia-resistant of the IEB suggests a strong link between HIF-1α and IEB, with notable therapeutic implications for IBD management.

Studies have indicated HIF-1α improves IBD outcomes by augmenting IEB function through various pathways. HIF-1α fortifies the IEB first by regulating cellular junctions, especially tight junctions, and IEC permeability (91–93). It then directly controls barrier-protective genes for maintaining and repairing intestinal barrier, including intestinal trefoil factor (ITF), cluster of differentiation 39 (CD39), CD73, vascular endothelial growth factor (VASP), multidrug resistance protein 1 (MDR-1), and integrins α2, α6 (34, 94–96). Additionally, HIF-1α sustains IEB performance by managing mucosal immune homeostasis (97, 98), and by influencing the gut microenvironment and metabolic pathways (34, 44, 99). Finally, enteric neurons and enteric glia in the enteric nervous system (ENS) are important regulators of IEB function and play a key role in maintaining IEB and resisting inflammation (100). Research indicates that HIF-1α is closely linked to the protective and death regulation mechanisms of enteric neurons. In trinitro-benzene-sulfonic acid (TNBS)-induced colitis in rats, glial cell line-derived neurotrophic factor (GDNF) protects enteric neurons from cell death due to metabolic challenges by activating HIF-1α and the REarranged during Transfection (RET) pathway (101). Although the exact mechanism of HIF-1α within the ENS remains unclear, the critical role of HIF-1α in preserving intestinal barrier function is undeniable.

#### 3.2.2 HIF-1α regulation of intestinal immunity

In the hypoxic environment of IBD, mucosal inflammation often involves extensive immune cells infiltration, which frequently determine the duration and intensity of the inflammatory response. The hypoxia signaling pathway centered on HIF-1 is present in nearly all cells, including immune cells, to help them adapt to hypoxic conditions (44, 57). Therefore, during the pathogenesis of IBD, HIF-1α inevitably interacts with immune cells, and under the conditions, the interactions between immune cells may also be mediated by HIF-1α. In IBD, HIF-1α plays a dual role in adaptive immunity. On the one hand, it modulates anti-inflammatory effects by regulating the functions and differentiation of T cells or B cells. HIF-1α is abundantly present in colonic T cells of IBD patients and colitis mice, restricting Th1 and Th17 cells activity and differentiation, while fostering regulatory T cells (Treg) differentiation and interleukin-10 (IL-10) release, thereby promoting T cells homeostasis and reducing gut inflammation (102–105). HIF-1α is also present in B cells, where it cooperates with phosphorylated signal transducer and activator of transcription 3 (p-STAT3) to regulate the transcription of CD11b, exerting an immunosuppressive effect to protect against IBD (106). It also enhances regulatory B cells (Breg) differentiation and IL-10 secretion to reduce intestinal inflammation (107, 108). On the other hand, HIF-1α may amplify inflammation by activating Th17 cells via ubiquitin-conjugating enzyme 9 (Ubc9) inhibition (109). The complex role of HIF-1α in adaptive immunity in IBD depends on its environment and regulatory mechanisms.

Recent research emphasizes the link between HIF-1α and innate immunity, especially its governance of macrophage functions crucial to IBD therapy (110). HIF-1α affects macrophage polarization, vital for immunological equilibrium in the gut. Macrophages exhibit a dual role, with M1 types inducing inflammation and M2 types exerting anti-inflammatory actions. These types are in a dynamic balance under physiological conditions, once disruption of this balance leads to inflammation. Mesenchymal stem cells, for example, encourage an anti-inflammatory state by shifting macrophages from M1 to M2 phenotypes through HIF-1α pathways, easing colitis (111–113). IBD medications like tiliroside, targeting the HIF-1α/glycolysis pathway (114), and spermidine, which boosts HIF-1α via reactive oxygen species (ROS)-activated Adenosine 5’-monophosphate (AMP)-activated protein kinase (AMPK) to favor M2 polarization (115), work by adjusting the M1/M2 balance. Dioscin’s anti-colitic effect also involves HIF-1α-mediated macrophage polarization (116). However, in IBD and murine colitis, hypoxia-driven HIF-1α in macrophages activates Wnt/mTOR pathways, disrupting autophagy in IEC (117). Since autophagy is key in IBD, regulating IEC metabolism, maintaining cell junctions, preventing IEC death, and aiding gut repair (118, 119), HIF-1α’s role in hypoxic immune cells may not uniformly benefit IBD pathology.

Dendritic cells (DC) are pivotal for intestinal immune balance, bridging innate and adaptive immunity. HIF-1α, abundant in DC, supports their function and adaption in hypoxia (57, 120). HIF-1α deletion in DC heightens pro-inflammatory factors and reduces Tregs, exacerbating intestinal inflammation (121). HIF-1α also interacts with neutrophils in IBD, where they serve ambivalent roles. Initially, they mitigate inflammation, but overactivation leads to barrier disruption and increased inflammation (122). Myeloid HIF-1α deficits encourage pro-inflammatory neutrophil and monocyte infiltration, hinder M2 polarization, and worsen colitis (66). Cyclosporine A eases severe UC by repressing sirtuin 6 (SIRT6)-driven neutrophil HIF-1α, enhancing glycolysis and tricarboxylic acid (TCA) cycle, restraining neutrophil overactivation, and preserving mucosal homeostasis (123). Additionally, advances in research on neutrophil extracellular traps (NET) in IBD suggests their dual role in inflammation (124), akin to HIF-1α, hinting at a potential hypoxia-driven HIF-1α-NET link in IBD pathogenesis worth further exploration. In summary, HIF-1α impacts intestinal immune equilibrium by modulating immune cells functions, thereby influencing IBD initiation and progression, though its precise role is modulated by variables like cell type, inflammation stage, and oxygen availability (**Table 1**).

**Table 1:** Role and mechanisms of HIF-1α in regulating immune cells in IBD.

Study setting	Animal models	Targets	Effects and mechanisms	References
in vivo and in vitro	CD45RB^high-^induced colitis	Treg	Hypoxia induces FoxP3 via HIF-1α in T cells, enhancing the abundance and functionality of Treg cells	(102)
in vivo、 in vitro and clinical samples	DSS-induced colitis、 TNBS- induced colitis、Citrobacter rodentium-induced infectious colitis	Th1、Th17	HIF-1α selectively induces IL-12p40 to inhibit the differentiation of Th1 and Th17 cells	(103)
in vivo and in vitro	DSS-induced colitis	Th17	Costunolide inhibits Th17 cell differentiation by suppressing HIF-1α-mediated glycolysis	(104)
in vivo and in vitro	DSS-induced colitis	CD4+ T Cells	HIF-1α in CD4+ T cells mediates the transcription of K^+^_2P_5.1, which plays a role in inducing Treg cells differentiation and IL-10 secretion	(105)
in vivo and clinical samples	Rag1^−/−^ mice	Th17	HIF-1α binds to the Ubc9 promoter, inhibiting Ubc9 and enhancing Th17 expression	(109)
in vivo and in vitro	DSS-induced colitis	B cells	HIF-1α synergizes with p-STAT3 to regulate transcription of CD11b in B cells, thus exerting an immunosuppressive effect	(106)
in vivo and in vitro	DSS-induced colitis	Breg	Mitochondrial oxidative phosphorylation regulates IL-10 production in Breg via control of HIF-1α and ERK signaling pathways	(107)
in vivo and in vitro	TNBS- induced colitis、 DSS-induced colitis	Macrophages	Mesenchymal stem cells promote M1 to M2 polarization via the HIF-1α/PI3K-γ or PHD2/HIF-1α pathways	(111–113)
in vivo	DSS-induced colitis、TNBS- induced colitis	Macrophages	Tiliroside reshapes M1/M2 balance via the HIF-1α/glycolysis pathway	(114)
in vivo and in vitro	DSS-induced colitis	Macrophages	Spermidine enhances mitochondrial ROS-activated AMPK, upregulating HIF-1α expression to induce M2 polarization	(115)
in vivo and in vitro	DSS-induced colitis	Macrophages	Dioscin mediates macrophages polarization by modulating the mTORC1/HIF-1α and mTORC2/PPAR-γ signaling pathways	(116)
in vivo、 in vitro and clinical samples	DSS-induced colitis	Macrophages	In hypoxic macrophages, HIF-1α induces Wnt1 expression and activates the Wnt and mTOR signaling pathways, thereby impairing IEC autophagy	(117)
in vivo	DSS-induced colitis	DC、Treg	HIF-1α in DC inhibits the production of pro-inflammatory factors and activates Treg, thus exacerbating intestinal inflammation	(121)
in vivo	DSS-induced colitis	Neutrophils、Ly6C monocytic cells、Macrophages	Myeloid HIF-1α deficiency increases the infiltration of pro-inflammatory neutrophils and monocytes, hindering macrophages polarization towards the M2 phenotype	(66)
clinical trial	–	Neutrophils	Cyclosporine A attenuates neutrophil hyperactivation through the SIRT6-HIF-1α-glycolysis axis	(123)

HIF-1α, hypoxia-inducible factor-1α; IBD, inflammatory bowel disease; Treg, regulatory T cells; FoxP3, forkhead box P3; DSS, dextran sulfate sodium; TNBS, trinitro-benzene-sulfonic acid; IL-12, interleukin-12; Ubc9, ubiquitin-conjugating enzyme 9; STAT3, signal transducer and activator of transcription 3; CD11b, cluster of differentiation 11b; Breg, regulatory B cells; ERK, extracellular regulated protein kinases; PI3K-γ, phosphoinositide 3 kinase-γ; PHD, prolyl hydroxylase enzymes; ROS, reactive oxygen species; AMPK, Adenosine 5’-monophosphate (AMP)-activated protein kinase; mTORC1, mammalian target of rapamycin complex 1; PPAR-γ, peroxisome proliferators-activatedreceptor; mTOR, mammalian target of rapamycin; IEC, intestinal epithelial cells; DC, dendritic cells; Ly6c, lymphocyte antigen 6c; SIRT6, sirtuin 6.

#### 3.2.3 HIF-1α modulates intestinal fibrosis

Intestinal fibrosis, a common complication of IBD, is primarily caused by chronic inflammation, with the pathogenesis mainly related to an excessive accumulation of extracellular matrix and an increase in the number of stromal-like cells in the submucosal layer (125). Previously considered irreversible and untreatable, this view of intestinal fibrosis has shifted due to recent studies. Extensive evidence has indicated that HIF-1α signaling plays a crucial role in various fibroses, including intestinal (17, 126–128). The autotaxin-lysophosphatidic acid (ATX-LPA) axis as a promising therapeutic target for CD-associated fibrosis, influencing fibroblast proliferation and differentiation, with HIF-1α modulating ATX expression (129). In IBD patients and colitis-induced mice, hypoxia alleviates fibrosis by inhibiting the transcription of pro-fibrotic genes (130). Betulinic acid hydroxamate, a PHD inhibitor targeting HIF, attenuates inflammation and fibrosis in colitis models, also aiding wound healing (131). Although evidence has supported HIF-1α’s antifibrotic effects, knowledge of its precise mechanisms is limited, and significant progress towards clinical application remains a lengthy process.

## 4 SCFAs in IBD

### 4.1 Basic overview of SCFAs in IBD

Growing research underscores the influence of gut microbiota and their metabolites, particularly SCFAs, on health and disease. SCFAs, as one of the gut microbiota’s main metabolite, are extensively confirmed to be a key contributor to the pathogenesis of IBD. IBD patients have fewer SCFAs-producing bacteria and lower fecal SCFAs levels than healthy individuals (132–134). Dietary fiber scarcity worsens colitis, while supplementation encourages SCFAs-producing bacteria and SCFAs levels in IBD, restoring gut homeostasis (135, 136). Notably, significant SCFAs concentration gradients exist along the gastrointestinal tract, with cecum and colon’s anaerobic conditions being prime for fermenting dietary fibers into SCFAs. Hence, the absence of the cecum decreases SCFAs-producing microbiota and SCFAs levels, intensifying inflammation (137). Understanding the role and related mechanisms of SCFAs in IBD is therefore crucial for the disease’s prevention and treatment.

### 4.2 The role and mechanism of SCFAs in IBD

#### 4.2.1 SCFAs modulate the intestinal barrier

The gut barrier, mainly composed of IEC, immune cells, and gut microbiota, encompasses mechanical, chemical, immune, and biological defenses, essential for protecting against harmful substances (138). Studies have indicated that SCFAs modulate IEB function through multiple pathways. SCFAs, particularly butyrate, serve as the main energy source for IEC, satisfying roughly 70% of colonic cells’ energy needs through mitochondrial β-oxidation, with the distal colon relying more heavily on butyrate than the proximal section (51, 139, 140). Besides, butyrate also enhances nutrient absorption in the gut by modulating NLRP3 pathway, providing further nourishment (141, 142). The energy from SCFAs is essential for regulating IEC activities such as proliferation, differentiation and apoptosis, which are critical for maintaining IEB integrity, intestinal energy metabolism, and immune regulation (22, 143). The “starving gut” hypothesis posits that IBD stems from mucosal malnutrition and energy deficiency associated with SCFAs scarcity, mainly manifesting as IEB defects and the persistence of the disease (139, 144, 145). Consequently, the energy provided by SCFAs is vital for the IEB function. Conversely, numerous perspectives critically refute the conventional view of butyrate as the first fuel of IEC, indicating that butyrate alone cannot fulfil the carbon needs for IEC synthesis, necessitating the involvement of DNA, RNA, proteins, and lipids. Hence, theoretically, butyrate may not be the sole carbon source for IEC proliferation (146). Moreover, several studies have shown that butyrate not only fail to promote IEC proliferation but may also inhibit it (147, 148), and even suppress the proliferation of colon cancer cells (149). The contradictions in the role of SCFAs in IEC proliferation might relate to the energy metabolism of different IEC types, such as undifferentiated versus differentiated cells, as not all IEC metabolize butyrate equally, with undifferentiated IEC preferring glucose over butyrate as a cellular fuel (150). Additionally, we believe that this might also be linked to the gut environment in various diseases and available energy sources for gut cells. Therefore, further research is necessary to elucidate the energy sources for IEC and the regulatory role of SCFAs in IEC proliferation.

SCFAs enhance gut barrier function by modulating microbiota composition and reducing luminal pH. The microbiota-driven bio-barrier is a crucial component of intestinal defenses. The increase of SCFAs significantly improve the structure of intestinal microbiota and accelerate the recovery of intestinal microflora, which is mainly manifested by increasing the number of beneficial bacteria while inhibiting harmful bacteria (151, 152). SCFAs reduce the colonic lumen pH and create a favorable acidic growth environment for probiotics such as Lactobacillus and bifidobacterium, thus maintaining the intestinal barrier function (153, 154). Besides, butyrate prevents colitis by modulating gut microbiota and reducing IgA-coated bacteria in the colon (155), and SCFAs inhibit the production of enterotoxin-producing bacteria, critical for protecting IEC and preventing intestinal diseases (156, 157). Additionally, due to their small molecular weight, SCFAs permeate IEB and exert antitoxin effects by modulating specific intracellular targets such as HIF-1, although the exact mechanisms remain unclear (156).

SCFAs also directly maintain IEB function by modulating tight junctions and mucous secretion. Tight junctions, vital for cellular polarity and permeability, consist of transmembrane proteins, cytoplasmic attachment proteins, and cytoskeletal proteins, with transmembrane proteins being most impactful (158). Their malfunction leads to compromised IEB (159). Occludin and claudins, especially claudins, are critical transmembrane proteins for tight junction (160). Studies have indicated SCFAs significantly upregulate tight junction proteins like zonula occludens-1 (ZO-1), occludin, and claudins, thus protecting IEB and attenuating inflammation (161, 162). The gut mucus layer, a physical barrier protecting IEC from harmful substances and the first line of defense for gut health, is mainly composed of mucins produced by goblet cells in IEC and nourishes the gut’s symbiotic flora (163–165). Therefore, mucus layer damage is linked to various diseases including IBD. SCFAs maintain the mucus barrier by regulating IEC mucin secretion. For instance, evidence has suggested that dietary fiber and SCFAs, particularly propionate and butyrate, promote the expression of mucins such as Mucin 2 (MUC2), enhancing the thickness of the intestinal mucus layer, thus alleviating gut inflammation and potentially reducing the risk of colitis associated colorectal cancer (CAC) (166–169). Moreover, mucins actively protect against gut infections by modulating antimicrobial peptide production, altering antigen-presenting cell activity, and enhancing bacterial clearance (170).

#### 4.2.2 SCFAs modulate intestinal immunity

In IBD, the extensive presence of immune cells in inflamed gut tissue is a key indicator of disease. Likewise, SCFAs are abundant in the intestinal environment and regulate the immune system. They affect adaptive immune cells, particularly CD4+ T cells, by modulating their activity, proliferation, differentiation, and intercellular homeostasis. SCFAs represented by butyrate inhibit Th17 activity and differentiation, while promoting Treg activity and differentiation, thereby restoring Th17/Treg immune homeostasis to alleviate IBD (171–175). Additionally, pentanoate promotes IL-10 expression and reduces IL-17A secretion by reprogramming T and B cell metabolism and epigenetics (176). Notably, SCFAs immune modulation appears concentration-dependent, with normal gut concentrations (10-100 mM) key for mucosal immunity. Low butyrate levels encourage anti-inflammatory forkhead box P3 (Foxp3) Treg differentiation, while high levels drive pro-inflammatory gene expression in Treg and T cells via histone deacetylases (HDAC) inhibition (177).

SCFAs also modulate innate immune cells like neutrophils (178), macrophages (179), and dendritic cells (180) to preserve gut immunity. In mouse models, dietary acetate reduces colitis by controlling neutrophil recruitment (178). NET are vital defenses produced by neutrophils at inflammation sites that capture and destroy pathogens, effectively controlling infections (181). Although direct links between SCFAs and NET formation in IBD are not well-established, research on neuroendocrine tumors shows that SCFAs induce NET in a pH-dependent manner (182). Thus, the potential link between SCFAs and NET in the pathogenesis of IBD warrants further research for validation. Macrophages show notable adaptability to the gut microbiome, typically without inciting inflammation. However, IBD or antibiotic abuse disrupts the gut microbiome and SCFAs, unbalancing macrophages and T cells, triggering cytokine release, which butyrate supplementation improves above situation (179). Additionally, butyrate suppresses M1 macrophage polarization to reduce inflammation, and even to inhibit CAC progression (141, 183, 184). Moreover, butyrate works with chemokine cytokine receptor 9 (CCR9)-recruited Myeloid-derived suppressor cells (MDSC) to alleviate DSS-induced colitis (185). MDSC originate from bone marrow and accumulate in IBD-compromised intestines, serving as a key IBD treatment target (186). Studies show MDSC curb intestinal inflammation by restraining T cells proliferation, natural killer cells (NK) cytotoxicity, and promoting anti-inflammatory macrophages phenotype (187, 188). However, MDSC have also been found to enhance inflammation by stimulating T cells like Th17 reflecting their diverse roles in IBD (186). Besides, invariant natural killer T cells (iNKT), linking innate and adaptive immunity, are modulated by SCFAs to regulate gut balance and inflammation by secreting IL-10 and inhibiting Th1 and Th17 (189). While debates over SCFAs’ role in gut immunity, their overall positive influence on IBD is evident. It is imperative to emphasize that SCFAs primarily modulate gut immunity by influencing cytokine production, migration, and functionality. In summary, SCFAs are integral to intestinal immunity and profoundly influence IBD pathology. Therefore, SCFAs-mediated immune modulation is pivotal for advancing IBD therapy, but it’s indispensable to consider SCFAs type, concentration, intestinal environment (e.g., pH) and target cells (**Table 2**).

**Table 2:** Role and mechanisms of SCFAs in regulating immune cells in IBD.

SCFAs	Study setting	Animal models	Target	Effects and mechanisms	References
Acetate、Butyrate	in vivo and in vitro	DSS-induced colitis	Th17、Treg	Acetate and butyrate activate PPARγ to inhibit Th17 differentiation and promote Treg differentiation, restoring the Th17/Treg balance	(171, 174)
Butyrate	in vivo	DSS-induced colitis、CDI-induced colitis	Th17	Butyrate inhibits Th17 activity via the SIRT1/mTOR pathway activation	(172)
Butyrate	in vivo	DSS-induced colitis	Th17、Treg	EcN-Sj16 suppresses Th17 differentiation and promotes Treg differentiation via the Ruminococcaceae/butyrate/retinoic acid axis, restoring the Th17/Treg balance	(173)
Acetate 、 Propionate	in vivo	DSS-induced colitis	Th17、Treg	Ento-A reshapes the gut microbiota to enhance acetate and propionate production, thereby inhibiting Th17 differentiation and promoting Treg differentiation to re-establish Th17/Treg balance	(175)
Pentanoate	in vivo	Rag1^+−/−^ mice	T cells、B cells	Pentanoate modulates T and B cells metabolism and epigenetic reprogramming to promote IL-10 expression and reduce IL-17A secretion	(176)
Butyrate	in vivo	DSS-induced colitis	CD4+T cells	Butyrate elevates T-bet and IFN-γ expression levels in acute colitis	(177)
Acetate	in vivo	DSS-induced colitis	Neutrophils	Dietary fiber mitigates intestinal inflammation by regulating neutrophil recruitment and infiltration via acetate	(178)
Butyrate	in vivo and in vitro	antibiotic-treated mice	Macrophages、T cells	Butyrate restores macrophages homeostasis and inhibits T-cells inflammatory cytokine production	(179)
Butyrate	in vivo and in vitro	DSS-induced colitis	Macrophages	Butyrate suppresses NLRP3 signaling and inhibits M1 polarization	(141)
Butyrate	in vivo、 in vitro and clinical samples	The CAC model induced by the AOM and DSS	Macrophages	Butyrate inhibits the NLRP3 signaling pathway and M1 polarization, reducing intestinal inflammation and limiting the progression of CAC	(183, 184)
Butyrate	in vivo	DSS-induced colitis	MDSC	Butyrate collaborates with CCR9-recruited MDSC to alleviate colonic inflammation	(185)
SCFAs	in vivo、 in vitro and clinical samples	IL10 mice	iNKT、CD4+ T cells	SCFAs enhance IL10 secretion by iNKT and inhibit the pathogenicity of T lymphocytes such as Th1 and Th17	(189)

SCFAs, short-chain fatty acids; EcN-Sj16, the genetically engineered Nissle 1917; Ento-A, the alcoholic extract of Periplaneta americana L; CAC, colitis associated colorectal cancer; T-bet, T-box expressed in T cells; IFN-γ, interferon γ; AOM, azoxymethane; MDSC, myeloid-derived suppressor cells; CCR9, chemokine cytokine receptor 9; iNKT, invariant natural killer T cells.

It is important to note that the pathways of SCFAs in modulating gut barrier and immune functions are often closely linked with G protein-coupled receptor (GPCR) and/or HDAC signaling, regarded as bridges between the host and gut microbiota. On the one hand, SCFAs modulate IEC and immune cell expression and function directly through activating GPCR (primarily GPR41, GPR43, and GPR109a) (190–195) and/or inhibiting HDAC (191, 196–200). On the other hand, SCFAs intervene in IBD by targeting downstream signaling cascades via these mechanisms, for instance, GPCR mediate SCFA-induced activation of mitogen-activated protein kinase (MAPK) (201, 202), nuclear factor kappa-B (NF-κB) (201), phosphoinositide 3 kinase (PI3K)/mTOR (203), NLRP3 (204), and STAT (203, 205) pathways, essential for the expression of key immune and inflammatory mediators. HDAC mediate SCFAs regulation of Toll-like receptor 4 (TLR4) (206) and NF-κB (192) signaling pathways. Notably, in IBD, the potential mechanism of SCFAs to regulate intestinal function is not limited to the above two pathways. SCFAs also influence intestinal function by acting on HIF signaling pathway, which is another key mechanism for SCFAs in physiological regulation.

## 5 HIF-1α mediates SCFAs regulation of IBD

### 5.1 HIF-1α mediates SCFAs regulation of intestinal hypoxia

The gut microbiota and hypoxia are closely linked, engaging in crosstalk within the host’s intestines. Microbial-derived SCFAs elevate IEC oxygen consumption to maintain intestinal hypoxia which activates and stabilizes HIF, triggering the adaptive cellular responses to hypoxia essential for intestinal function and pathology (54). HIF-1α in the gut induces pyruvate dehydrogenase kinases (PDK or PDHK) expression, such as PDK1 and PDK3, inactivating the pyruvate dehydrogenase complex (PDC) and blocking pyruvate’s conversion to acetyl-CoA for the TCA cycle. Thus, colonocytes depend on SCFAs β-oxidation, especially of butyrate, for acetyl-CoA production, a highly oxygen-consuming process (207). Moreover, butyrate directly inhibits HDAC, inducing PDK1-4 expression (208). In short, SCFAs, particularly butyrate, directly or indirectly promote PDK activation to switch acetyl-CoA generation from glycolysis to SCFAs oxidation, key in SCFAs’ regulation of HIF-1α to preserve IEC energy supply and gut hypoxia. Thus understanding the physiological regulation of HIF-1α by SCFAs in IBD could inform prevention and treatment strategies for the condition.

### 5.2 HIF-1α mediates SCFAs regulation of the intestinal barrier

**Figure 2:**
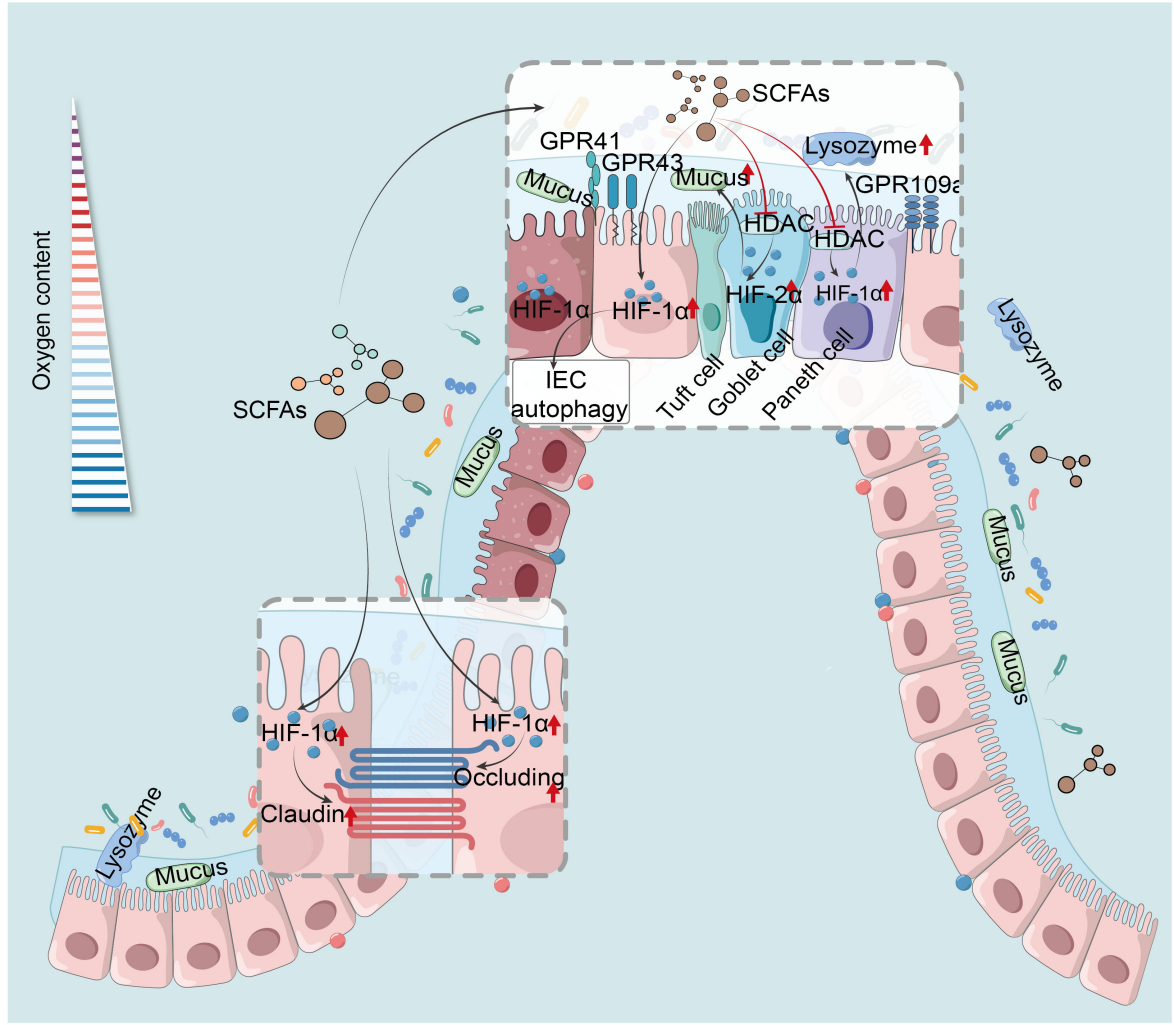
HIF mediates SCFAs regulation of the intestinal barrier and IEC autophagy. SCFAs reinforce IEB by activating HIF-1α in IEC, heightening tight junction proteins, and curtailing permeability and bacterial translocation (93, 210). Their anti-inflammatory action stabilizes HIF-1α via HDAC inhibition, boosting lysozyme activity (211). SCFAs activate HIF-2α, enhancing goblet cell MUC2 secretion and mucosal defense (215). SCFAs induce autophagy in IEC by up-regulation of HIF-1α (76). HIF-1α, hypoxia-inducible factor-1α; SCFAs, short-chain fatty acids; GPR41, G protein-coupled receptor 41; HDAC, histone deacetylases; IEC, intestinal epithelial cells.

SCFAs trigger HIF-1α in IEC, essential for maintaining IEB function and gut homeostasis. They enhance IEC survival, metabolism, and tight junction through HIF-1α activation, protecting the IEB from inflammatory and infectious damage (54, 209). For example, butyrate enhances IEB function by activating HIF-1α in IEC to upregulate tight junction protein expression, reducing barrier permeability and bacterial translocation, thereby mitigating colonic damage caused by colitis or Clostridioides difficile infection (CDI) (93, 210). However, butyrate’s precise mechanism in HIF-1α modulation remains unclear, suspected to involve physiological hypoxia. Additionally, butyrate stabilizes HIF-1α via HDAC inhibition, enhancing antimicrobial lysozyme activity (211). Research has revealed that the butyrate-producing bacterium Faecalibacterium prausnitzii can modulate HIF-1α to enhance IEC IL-18 expression, potentially aiding in mucosal healing in IBD, offering new insights into HIF-1α-mediated SCFA regulation of intestinal mucosal repair and IEB function maintenance (212). Antibiotic-induced experiment has suggested butyrate stabilizes colonic HIF-1α in mice by inhibiting PHD and increasing 2-oxoglutarate levels, beneficial for gut homeostasis (55). Moreover, SCFAs and HIF-1α play roles in VDR-mediated mucosal barrier protection under hypoxia. Moderate hypoxia induces HIF-1α-VDR promoter binding, enhancing barrier function, with VDR deficiency altering gut microbiota and reducing SCFAs-producing bacteria (61). The role of SCFAs in HIF-1α-regulated VDR signaling under hypoxia needs further investigation. Additionally, SCFAs stimulate mucin expression, critical for barrier function, with HIFs, particularly HIF-1α, being key mucin regulators (213). In asthma studies, butyrate affects HDAC1 through PI3K/Akt/HIF-1α/VEGF pathways, moderating mucus secretion and inflammation (214). However, current IBD research lacks understanding of the synergistic impact of SCFAs and HIF-1α on mucin. While HIF-2α is pivotal in propionate-induced mucin expression, promoting MUC2 secretion from goblet cells, no HIF-1α involvement has been observed, as propionate’s HIF-2α activation for MUC2 production may suppress HIF-1α influence (215). This aligns with findings that increased HIF-1α suppresses HIF-2α and HIF-3α, and vice versa (58). In conclusion, HIF-1α maintains stable expression in IEC under physiological hypoxia, with SCFAs, especially butyrate, being essential for this stability. Therefore, butyrate holds substantial promise as endogenous PHD inhibitors to stabilize HIF-1α and maintain IEB function (**Figure 2**).

### 5.3 HIF-1α mediates SCFAs regulation of intestinal immunity

**Figure 3:**
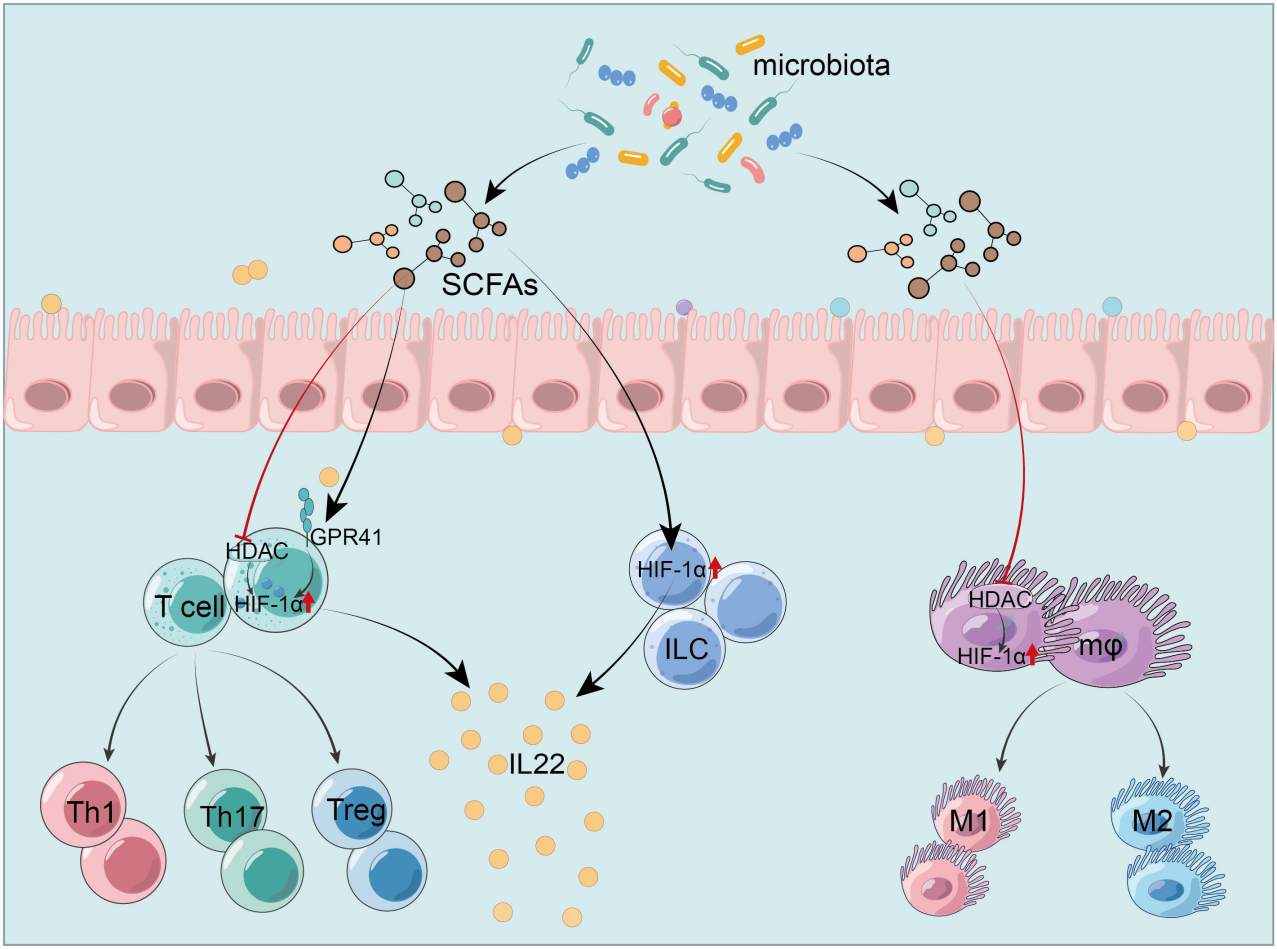
HIF-1α mediates SCFAs regulation of the intestinal immunity. SCFAs modulate HIF-1α to activate ILC and increase IL-22 production (216). They trigger IL-22 production in CD4+ T cells and ILC by activating GPR41 and inhibiting HDAC, which upregulates HIF-1α (217). SCFAs enhance the antibacterial activity of macrophages by inhibiting HDAC and up-regulating HIF-1α (218). HIF-1α, hypoxia-inducible factor-1α; SCFAs, short-chain fatty acids; GPR41, G protein-coupled receptor 41; HDAC, histone deacetylases; IEC, intestinal epithelial cells; ILC, innate lymphoid cells; Treg, regulatory T cells; IL-22, interleukin-22.

In IBD, SCFAs stabilize HIF-1α, modulating immune cells function in the gut. José Luís’s team demonstrated that butyrate triggers the HIF-1α-IL-22 axis in ILC3s, reducing CDI-triggered gut inflammation and reinforcing IEB integrity (216). It also upregulates HIF-1α and Aryl hydrocarbon receptor (AhR) via GPR41 activation and HDAC inhibition, promoting IL-22 in CD4+ T cells and ILCs (217). This mechanism involves butyrate’s histone modifications, which increase HIF-1α binding to the IL-22 promoter, augmenting IL-22 expression (217). These studies provide new insights into SCFAs’ role in anti-inflammatory mediator production. Furthermore, butyrate enhances macrophage antibacterial activity by upregulating HIF-1α through HDAC inhibition without GPCR involvement, enhancing antimicrobial components’ expression like lysozyme and ROS, and raising oxygen consumption by about 50%. However, inhibition of HIF-1α could block the effects of butyrate (218). In research on Streptococcus pneumoniae, acetate boosts macrophage glycolysis, activating HIF-1α and NLRP3 inflammasome, triggering IL-1β release and enhancing nitric oxide (NO) synthesis, which in turn improves macrophage bactericidal function through NO reliance (219). Hence, the glycolysis-HIF-1α pathway is pivotal for SCFAs-mediated macrophages cytotoxicity regulation. However, focused research on HIF-1α in SCFAs-modulated colitis macrophages remains scarce, representing a promising research avenue, especially given its importance in conditions like pneumonia. Additionally, pentanoate facilitates iron uptake in intestinal cells, thereby augmenting HIF-2α expression and inducing c-Maf production, which is crucial for intestinal Treg differentiation and homeostasis (220). Notably, c-Maf typically acts as a positive regulator of IL-10 across various immune cells. However, whether pentanoate prompts Treg or other immune cells to secrete IL-10 was not clarified and warrants further study (**Figure 3**).

SCFAs not only modulate immune cells directly but also influence downstream inflammatory pathways through HIF-1α. Butyrate suppresses HDAC and activates the p-glycogen synthase kinase-3β (GSK-3β)/β-catenin/HIF-1α/NF-κB pathway, reducing inflammation in sleep deprivation-induced colitis mice (221). In human peripheral blood mononuclear cells, butyrate mitigates inflammation by blocking TLR4 signaling without affecting HIF-1α expression and the HIF-1α/HIF-2α ratio. In contrast, caprate, a medium-chain fatty acid (MCFA), activates inflammation via TLR2, reduces IL-10 and HIF-1α, and increases HIF-2α transcription, altering the HIF-1α/HIF-2α ratio (222). The differential immunomodulatory effects of fatty acids may relate to their origins or structures, with SCFAs being endogenous and MCFAs exogenous from diet. Structurally, SCFAs and MCFAs differ by a few carbon atoms. The variance in HIF-1α and HIF-2α expressions might be a compensatory response to inflammation induced by caprate. Besides, high concentrations of acetate in organoid-derived epithelial monolayers from UC patients exhibits anti-inflammatory effects and reinforces barrier function, concurrently elevating HIF-1α levels, though mechanisms are uncertain (223). Notably, acetate lowers IL-10 levels, which contrasts with the view that SCFAs promote IL-10 expression, suggesting that SCFAs type, concentration, or cell-type differences might play a role. Overall, SCFAs-targeted HIF-1α modulation offers a strategy to combat intestinal inflammation. The promotion of HIF-1α in IEC by SCFAs is largely linked to physiological hypoxia, and the HIF pathway is one of the most important pathways to communicate between the host and the intestinal microecology in addition to GPCR and HDAC signaling.

### 5.4 HIF-1α mediates SCFAs regulation of autophagy

SCFAs activate HIF-1α to induce autophagy in IEC, beneficial for suppressing intestinal inflammation in IBD. In mouse IEC, butyrate eases DSS-induced colitis by upregulating HIF-1α to modulate autophagy and gut microbiota; however, HIF-1α deficiency lowers intestinal butyrate concentration and autophagy levels to increase susceptibility to colitis (**Figure 2**) (76). The reduced autophagy from HIF-1α deficiency may relate to the disruption of gut physiological hypoxia. Hypoxia or increased oxygen consumption also induces IEC autophagy (62), highlighting HIF-1α’s role in SCFAs-mediated autophagy regulation. Besides, SCFAs and HIF-1α are closely linked to PTEN induced putative kinase 1 (PINK1), an ubiquitin kinase essential for mitochondrial autophagy (224). In CAC models, PINK1 hampers tumor growth by activating p53 and HIF-1α/PDHK1/PDHE1α pathways, reducing acetyl-CoA production; contrastingly, acetate exposure increases acetyl-CoA in tumors, counteracting PINK1’s anti-tumorigenic effects (225). This indicates that the roles of SCFAs and HIF-1α in CAC and IBD are not constant and necessitate tailored analysis for future therapeutic applications.

## 6 Conclusion and outlook

In recent years, the intricate interplay among hypoxia, gut microbiota, and intestinal immunity has sparked significant interest among researchers. Substantial evidence has suggested that the dysregulation of gut microbiota and its metabolites, SCFAs, under hypoxic conditions, serves as a pivotal pathological feature of IBD and is a key contributor to the dysfunction of IEB and immune system. HIF-1α stands at the core of this complex ecological network, maintaining intestinal homeostasis. However, reviews on the role and mechanisms of HIF-1α-mediated SCFAs regulation in IBD under hypoxic conditions are scarce. Therefore, this review summarizes the roles and mechanisms of SCFAs in stabilizing HIF-1α to regulate IEB function and intestinal immunity under hypoxia, findings that are crucial for elucidating the pathophysiology of IBD and its prevention and treatment.

Hypoxia, a common physiological state in the host gut, provides a stable environment essential for maintaining cellular metabolism, immune homeostasis, and microbiota balance, with HIF-1α being the key regulatory factor under these conditions. HIF-1α and SCFAs preserve IEB function through regulating tight junctions, mucous secretion, and microbial homeostasis. Moreover, they modulate adaptive and innate immune cells homeostasis to suppress colonic inflammation, closely related to T-cells differentiation and macrophage polarization. Notably, SCFAs are closely linked to HIF-1α, playing a pivotal role in hypoxia-induced HIF-1α stabilization, and they synergistically modulate IBD pathogenesis. HIF-1α mediates SCFAs’ regulation of IEB function, intestinal immunity, and autophagy, a key mechanism in SCFAs’ therapeutic potential for IBD. Certainly, GPCR and HDAC are also significant pathways through which SCFAs regulate HIF-1α function. However, studies on the role of hypoxia/HIF-1α or SCFAs in IBD present contradictory conclusions, possibly related to factors such as colitis models, SCFA types and concentrations, gut environment (hypoxia levels, pH), and cell phenotype heterogeneity during experimentation. Considering the complexity of the gut environment and the significant impact of cellular phenotypic heterogeneity on IBD, current research challenges in elucidating the specific pathways and mechanisms by which HIF-1α and SCFAs regulate IBD. Therefore, future studies should focus on interdisciplinary collaboration, utilizing genomics, transcriptomics, and metabolomics, to comprehensively and multidimensionally explore the impact of these factors on the IEB and gut immunity, as well as their roles in different IBD stages. Moreover, single-cell RNA sequencing can reveal cellular phenotypic heterogeneity in the human gut, providing insights directly relevant to human diseases. Currently, animal models, mainly chemically induced such as DSS and TNBS, fail to fully replicate the intestinal characteristics and environment of human IBD. The development and adoption of animal models that closely mirror human physiological and pathological features, including gut microbiome composition, immune responses, and epithelial barrier functions, are essential. Genetic engineering can create models expressing or suppressing genes involved in human IBD, offering insights into underlying molecular pathways. If possible, future research should also more extensively incorporate data from human subjects, including using human intestinal organoids for a more accurate gut epithelial model and analyzing tissue samples from IBD patients.

Though the review presents SCFAs’ regulatory effects on HIF-1α in IBD and their proven therapeutic value in experimental studies, clinical treatments designed to boost SCFAs synthesis in the body, like dietary fiber, probiotics, and polyphenols, fall short of expectations due to human physiological complexities. Research shows that while widespread microbial dysregulation in the intestines of adult IBD patients during remission, their capacity to ferment fiber and produce SCFAs is comparable to healthy individuals (226). Merely supplementing dietary fibers or SCFAs-producing probiotics seems insufficient to correct the gut dysbiosis in IBD. Moreover, despite the regulatory importance of SCFAs in gut homeostasis and health, excessive SCFAs, particularly butyrate, in both the gut and blood disrupt gut ecology, hinder anti-cancer treatment sensitivity, and promote a tumor-friendly microenvironment, detrimental to cancer patients (227, 228). This negative impact could be due to SCFAs levels in the gut surpassing the host’s threshold, which potentially leading to pro-inflammatory and even carcinogenic effects, offering insight into inconsistent results in gut immunity research. Also, with the diverse and sometimes antagonistic interactions among SCFAs types, it’s unclear whether single or multiple SCFAs supplementation is beneficial. Therefore, it becomes necessary to develop clinical methods for the quantitative detection of SCFAs in intestinal cells and precise targeted drug delivery, which may become a focal point in future clinical therapeutic research on SCFAs. Additionally, HIF-1α plays a pivotal role in cellular adaptation to hypoxia, maintaining gut homeostasis and enhancing SCFAs functions, making it a vital target for IBD prevention and treatment. Acetate, a key component of SCFAs and an endogenous inhibitor of PHD stabilizing HIF-1α expression, holds significant implications as a therapeutic target for IBD.

## Author contributions

JX: Conceptualization, Visualization, Writing – original draft. XG: Writing – review & editing. ZW: Conceptualization, Supervision, Validation, Writing – review & editing.
